# Preclinical Application of Computer-Aided High-Frequency Ultrasound (HFUS) Imaging: A Preliminary Report on the In Vivo Characterization of Hepatic Steatosis Progression in Mouse Models

**DOI:** 10.3390/jimaging11100369

**Published:** 2025-10-17

**Authors:** Sara Gargiulo, Matteo Gramanzini, Denise Bonente, Tiziana Tamborrino, Giovanni Inzalaco, Lisa Gherardini, Lorenzo Franci, Eugenio Bertelli, Virginia Barone, Mario Chiariello

**Affiliations:** 1 Institute of Clinical Physiology, National Research Council, 53100 Siena, Italy; giovanniinzalaco@cnr.it (G.I.); lisa.gherardini@cnr.it (L.G.); lorenzofranci@cnr.it (L.F.); mario.chiariello@cnr.it (M.C.); 2 Core Research Laboratory (CRL), Istituto per lo Studio la Prevenzione e la Rete Oncologica (ISPRO), 53100 Siena, Italy; 3 Institute of Crystallography, National Research Council, Montelibretti Division, 00010 Rome, Italy; matteo.gramanzini@cnr.it; 4 Department of Life Sciences, University of Siena, 53100 Siena, Italy; denise.bonente2@unisi.it (D.B.); tiziana.tamborrino@student.unisi.it (T.T.); 5 Department of Molecular and Developmental Medicine, University of Siena, 53100 Siena, Italy; eugenio.bertelli@unisi.it (E.B.); virginia.barone@unisi.it (V.B.)

**Keywords:** metabolic dysfunction-associated steatotic liver disease, animal model, diet manipulation, C57Bl/6J mouse, genetically engineered mice, high frequency ultrasound imaging, computer-aided diagnosis

## Abstract

Metabolic dysfunction-associated steatotic liver disease (MASLD) is one of the most common chronic liver disorders worldwide and can lead to inflammation, fibrosis, and liver cancer. To better understand the impact of an unbalanced hypercaloric diet on liver phenotype in impaired autophagy, the study compared C57BL/6J wild type (WT) and MAPK15-ERK8 knockout (KO) male mice with C57BL/6J background fed for 17 weeks with “Western-type” (WD) or standard diet (SD). Liver features were monitored in vivo by high-frequency ultrasound (HFUS) using a semi-quantitative and parametric assessment of pathological changes in the parenchyma complemented by computer-aided diagnosis (CAD) methods. Liver histology was considered the reference standard. WD induced liver steatosis in both genotypes, although KO mice showed more pronounced dietary effects than WT mice. Overall, HFUS reliably detected steatosis-related parenchymal changes over time in the two mouse genotypes examined, consistent with histology. Furthermore, this study demonstrated the feasibility of extracting quantitative features from conventional B-mode ultrasound images of the liver in murine models at early clinical stages of MASLD using a computationally efficient and vendor-independent CAD method. This approach may contribute to the non-invasive characterization of genetically engineered mouse models of MASLD according to the principles of replacement, reduction, and refinement (3Rs), with interesting translational implications.

## 1. Introduction

Metabolic dysfunction-associated steatotic liver disease (MASLD), previously known as non-alcoholic fatty liver disease (NAFLD) [1], is one of the most common chronic liver disorders globally, and can lead to inflammation, fibrosis, and liver cancer [2]. The most common form of MASLD, simple steatosis, may be reversible in the early stages. Therefore, its timely and sensitive identification and monitoring are essential to avoid progression to chronic liver damage through preventive and therapeutic interventions. Importantly, the association between MASLD, metabolic syndrome, and cardiovascular risks is increasingly recognized [3,4]. Fat accumulation in the liver is often associated with hyperlipidemia, hyperglycemia, hyperinsulinemia, and insulin resistance, as well as increased caloric intake and overweight, leading to an imbalance in the hepatic management of lipid homeostasis.

Mouse models can significantly improve the study of the pathogenetic mechanisms of MASLD, the identification of susceptibility genes, and novel therapeutic targets [4]. Several mouse models reproduce MASLD at different degrees of severity, mainly through dietary and/or genetic intervention [4,5]. None of them fully recapitulate the complex pathophysiology of human steatosis, in part due to species-specific differences in liver biology, and there is a lack of consensus regarding the optimal model [4,6]. Methionine-choline deficient diet (MCD) and Western-type diet (WD) mice represent the most widely used mouse models. The choice of the appropriate model depends on the specific research question, considering its advantages and shortcomings. Models with advanced liver disease are better suited to test treatments targeting inflammation, fibrosis, and tumor complications beyond steatosis, but may miss early therapeutic opportunities and are unlikely to fully replicate later stages of human disease [6]. In particular, mice fed MCD have been criticized for failing to properly replicate metabolic risk factors associated with human liver disease and for showing differences in metabolic pathways [4]. Currently, mouse models using WD with a fat, carbohydrate, and cholesterol content most similar to the unbalanced human diet in Western countries are considered the most accurate models for modeling the etiology, metabolism, and histological basis of human MASLD from early to advanced stages of the disease, including steatohepatitis [4]. In particular, long-term WD (12–16 weeks) can lead to mild inflammation and fibrosis, better fitting the chronic progression of human liver disease and providing a more comprehensive understanding of its different forms in patients [4]. Key factors for more clinically relevant mouse models also include the combination of diet-induced and genetically modified models and the implementation of methods to monitor the temporal evolution of hepatic steatosis.

These models are valuable for testing preventive therapies primarily against simple fatty liver disease or treatments that act during the early and more reversible stages of this disease. At the same time, experimental procedures that mirror clinical practices, such as the use of noninvasive imaging techniques or standardized, peer-reviewed histological scores, may help improve the translational value of research and refine methods according to the 3Rs [4].

In the clinical setting, liver biopsy is the gold standard for the diagnosis of early stages of hepatic steatosis, but its invasiveness hinders its repeated use for disease and treatment monitoring, and the procedure carries an inherent risk of sampling errors [7]. Computed tomography (CT) and magnetic resonance imaging (MRI) are reliable imaging modalities for evaluating MASLD, but they have some limitations, such as the use of ionizing radiation, high exam costs, and potential imaging artifacts that limit their widespread applicability for patient examination. Alternatively, ultrasound (US) is the preferred imaging modality to diagnose NAFLD in the clinical setting, because it is feasible, safe, and cost-efficient to study the liver. Visual inspection of grayscale B-mode US images is by far the first-line diagnostic method used clinically for the investigation of hepatic steatosis. US shows a correlation with clinical risk factors of MASLD, as well as a sensitivity of 84.8% and a specificity of 93.6% in detecting moderate to severe steatosis [8,9]. However, conventional US (CUS) qualitative assessment of pathological changes in the liver parenchyma, such as brightness, coarseness, or heterogeneity, depends on both the operator’s experience and the scanner performance. Furthermore, CUS has a low intrinsic sensitivity and specificity in diffuse liver diseases, making it difficult to distinguish between fatty liver and fibrosis and detect mild hepatic fatty infiltration (<30%) [10,11]. Recent studies have addressed these limitations by focusing on quantitative US (QUS) approaches to characterize and classify hepatic steatosis. Indeed, QUS analysis of hepatic steatosis in patients has been shown to be more consistent across operators with different expertise, providing a non-invasive and relatively inexpensive method to assess and monitor MASLD [12]. A common semiquantitative measure is the hepatorenal index (HRI), which is widely implemented in commercial software for clinical US scanners. Nevertheless, concomitant renal disease could influence the validity of HRI by altering the brightness of the renal parenchyma, making a functional evaluation of the kidney advisable. The urgent need to identify a practical and routine method to assess the early stages of MASLD in humans, with greater sensitivity than blood tests, prompted the first studies using QUS in murine models. Liver attenuation and backscatter coefficients were determined in twenty male C57BL/6J mice fed a “Paigen-like” high fat diet for 4 weeks [13]. Compared to five controls, mice fed a high fat diet developed steatosis at experimental endpoint, showing a statistically significant correlation between QUS and ex vivo histological grading measures. However, the Paigen-like diets do not properly mimic the composition of the human WD and induce severe liver alterations in mice, raising further ethical concerns about animal welfare. Moreover, accurate measurement of US attenuation and backscatter coefficients requires specialized tools and algorithms, not readily accessible in standard US systems. Translationally, histologic examination is the current reference method in preclinical research for diagnosing hepatic steatosis, but requires animal sacrifice, increasing the number of subjects needed to monitor disease progression or therapeutic response at serial time points. Non-invasive high-frequency US (HFUS) imaging could significantly contribute to validate study hypotheses in animal models, allowing a longitudinal evaluation of the same subject, thus reducing both biological variability and the number of animals killed unnecessarily, in accordance with the 3Rs principle. Similarly to humans, conventional HFUS is effective for the diagnosis of advanced hepatic steatosis in mouse models, but presents challenges in detecting early or mild hepatic steatosis due to subtle alterations or technical difficulties, which may require complementary strategies to optimize disease assessment.

Sparse reports have proposed improved semi-quantitative HFUS approaches in rodent models. The progression of hepatic steatosis was monitored in rat models by the liver–renal cortex echogenicity ratio, showing a high correlation with the moderate/severe histopathological grading of fatty liver disease (sensitivity: 90%; specificity: 100%) [14,15,16]. Similarly, our research group has demonstrated that an integrated approach combining qualitative and parametric HFUS analyses of the liver in C57BL/6J mice fed a WD was able to detect longitudinal changes in liver echogenicity and echotexture, including an equal or higher brightness of the renal cortex [17]. Indeed, the liver–renal cortex echogenicity ratio showed a higher trend in db/db mice compared to C57BL6 controls [18]. More sophisticated methods have been used in mouse models, such as US-induced thermal strain imaging [19], US molecular imaging using microbubbles that target the fatty acid translocase CD36 and markers of liver inflammation [20], and Shear Wave Elastography [21]. However, these techniques require vendor-dependent software and specialized skills, labor-intensive procedures, and expensive equipment that is not widely available, hindering their practical and widespread use at high throughput, compared to other conventional B-mode image-based QUS techniques. Overall, the results described come from the examination of different mouse strains and sexes, and different diet compositions and durations, mainly in cross-sectional studies at advanced stages of the disease. Thorough standardization of methods in the workflow, based on the up-to-date literature on the topic, is therefore of relevance. Recently, the progress in artificial intelligence (AI) systems has promoted their use for the enhanced extraction of quantitative data within US images. Computer-aided diagnosis (CAD) can help to identify complex disease-related patterns early that are difficult to recognize with traditional methods. In this perspective, radiomics could not simply automate the diagnostic process, but complement existing methods with further optimized data.

Although US-based texture analysis has been implemented to a limited extent compared to CT and MRI, this emerging technique appears promising for identifying imaging features that distinguish healthy from pathological liver parenchyma. Various technical factors could have a great influence on the results, including random variations in subjects. Furthermore, US images may have a relatively low signal-to-noise ratio (SNR) due to artifacts related to motion, respiration, or intestinal gas, increasing the technical challenges of reliably calculating quantitative image features [22].

To our knowledge, the applications of AI in liver US imaging in preclinical models are limited. Reviewing the recent literature on this topic, few reports were found that focus primarily on liver fibrosis or the assessment of tumor lesions [23]. A CAD approach has been proposed to characterize steatosis on HFUS images of ex vivo mice livers [24]. In particular, seven selected features were extracted from the B-mode liver images of C57B6 and ob/ob mice, with efficient processing time and with an accuracy of 85.74%, as well as a sensitivity of 84.4%, and a specificity of 88.5% [24].

Overall, the limitations of the existing literature on this specific topic mainly include the use of unbalanced diets (which raise both scientific and ethical concerns), single image post-processing methods, complex/expensive imaging techniques, or data collection at a single time point. Furthermore, there is a lack of studies applying texture analysis to in vivo HFUS images of the murine steatotic liver. Our preclinical study aimed to explore the potential of a simple, reliable, refined methodology integrating CUS with different US imaging computerized analysis (USICA) approaches for a longitudinal monitoring of the early stages of hepatic steatosis in living C57BL/6J wild type (WT) mice and knock out (KO) mice for MAPK15-ERK8 genes in this genetic background, fed standard and Western-style diets, with histology as gold standard.

Our research group has conducted a comprehensive in vivo and in vitro characterization of the involvement of the MAPK15-ERK8 gene in the regulation of hepatocyte lipid homeostasis [25]. Few data are available in the literature on the effects of reduced hepatic autophagy in a genetically engineered mouse model (GEMM), which suggests a role in the pathogenesis of liver diseases related to the fat accumulation and inflammation [26,27,28]. To the best of our knowledge, a non-invasive investigation tracking in vivo the early liver alterations related to dietary intervention (DI) and/or impaired autophagy in murine models by integrated HFUS approaches represents a novel contribution in this field of research. The proposed methodology could be useful for the identification and quantification of different stages of hepatic steatosis in mouse models and predicting treatment response, with the advantages of accessibility, limited computational capabilities, and time required.

The tested mouse models are highly translatable, based on a high-fat, high-sugar diet that closely resembles the typical diet of Western societies, as does the applied diagnostic paradigm, including in vivo imaging parameters commonly employed in clinical settings for the routine assessment of liver diseases, and the matching of imaging data with blood biomarkers and histology.

In accordance with the 3Rs, an optimized HFUS methodology supports further refinement of disease progression monitoring in mouse models and reduction in the number of animals that need to be euthanized at different experimental time points, as well as maximizing the information obtainable from each animal.

## 2. Materials and Methods

### 2.1. Compliance with Ethical Standards

Animal experimentation was conducted in compliance with the European and national laws (Directive 2010/63/EU; D.L. 26/2014). The Animal Welfare Board of the Fondazione Toscana Life Sciences (FTLS) and the Italian Ministry of Health have approved this research project (authorization numbers: 175/3 March 2021; 175/17 February 2023). This study followed “The Animal Research: Reporting of In Vivo Experiments” (ARRIVE) and the National Institutes of Health (NIH) guidelines. The datasets used and/or analyzed during the current study are available from the corresponding author on reasonable request.

### 2.2. Study Design and Animals

We conducted a pilot study that included clinical and laboratory phenotyping of mice and a retrospective review of HFUS images for the implementation of USICA. The primary objective was to improve the identification of hepatic steatosis in mouse models by imaging markers, using histological classification as a reference for confirmation of MASLD. As imaging refinement, CUS scoring, and parametric analysis have been combined with HFUS texture analysis. The analyzed dataset includes US images from 23 male C57BL/6J WT mice and 22 male KO mice. Inbred C57BL/6J (JAX stock #000664) were obtained from Jackson Laboratory (Bar Harbor, ME, USA) via Charles River (Calco, LC, Italy). MAPK15-ERK8 KO mice were obtained from Taconic Biosciences (Hudson, NY, USA) and the originally hybrid B6; 129S-MAPK15/ERK8^−/−^ mice were transferred on a C57BL/6J background by backcrossing for at least 10 generations at the Centro di Biomedicina Sperimentale (CBS, Pisa, Italy). The experimental procedures were performed at the FTLS animal facility (Siena, Italy). Seven-week-old mice were group-housed (maximum four mice/cage, except one aggressive male mouse) under standard conditions (20–23 °C, 12 h light/12 h dark cycle) and with ad libitum access to water and standard diet (SD, 3% fat, 4RF21, Mucedola^®^, Settimo Milanese, Italy; Kcal 18.5% from protein; 3% from fat; 53.5% carbohydrates, 3% sucrose; kcal/g 3.150). Overall, based on the genotypes and DI, mice were divided into 4 groups: 7 WT and 7 KO male mice were fed SD, while 16 WT and 15 KO male mice were fed WD (0.2% cholesterol and 21% butter, Western U8958 version 35, SAFE^®^, France; Kcal 14,4% from protein; 38.1% from fat; 47% carbohydrates, 33% sucrose; kcal/g 4.2594) from 8 to 24 weeks of age. For practical reasons, we simultaneously examined a batch of animals comprising 8 WT males and 8 KO males fed WD. Subsequently, we examined two additional batches, each comprising at least 7 WT and 7 KO males fed the same diet (SD or WD), at two separate time periods.

### 2.3. Research Clinical Monitoring

To assess the impact of caloric intake on the change in body weight (BW, g) from baseline (BW measured at the beginning of the eighth week of life, before the start of the experiments), we measured these parameters weekly throughout the duration of the study.

Serum biochemical tests provide information on liver and metabolic dysfunction. To assess the impact of DI on blood biochemistry, non-fasting glucose, cholesterol, and triglycerides were measured at 8 (baseline, pre-diet) and 16 weeks of age in isoflurane-anesthetized mice (3% isoflurane + 2 Lt/min oxygen). At the experimental endpoint (24 weeks of age), mice were fasted for 3 h starting at 8:00 AM, then anesthetized as described above, and blood was collected through cardiocentesis before euthanasia for clinical biochemistry. Details about this experimental setup have been previously published [18].

### 2.4. Imaging

#### 2.4.1. Ultrasound Acquisition

**Figure 1:**
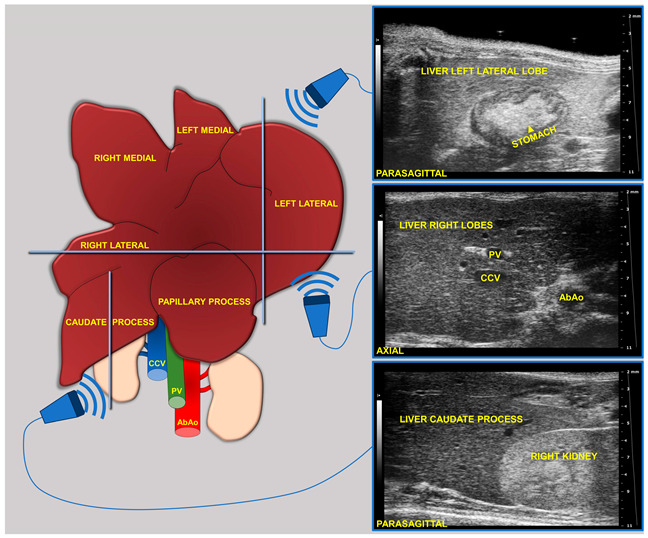
Schematic of the two-dimensional (2D) B-mode US imaging acquisition for murine liver.

During imaging sessions, body temperature, heart, and breath rates were monitored and normothermia was maintained using a heated platform and an infrared lamp. Mice were anesthetized with inhalant gases (induction chamber: 4% isoflurane plus 2 Lt/min oxygen; maintenance with nose cone: 1.5–1.8% plus 2 Lt/min oxygen). Subsequently, the mice were placed in a supine position and a coupling gel was applied to the trichotomized skin. US two-dimensional (2D) B-mode images of the liver of mice (left, right, and caudate lobes, sagittal and axial planes) at 8 (baseline, pre-diet), 16, and 24 weeks of age were acquired in real time by a veterinary radiologist with 17 years of experience (SG) (Figure 1). A dedicated HFUS equipment was used (Vevo 2100, FUJIFILM VisualSonics Inc., Toronto, ON, Canada; MS550 transducer: central frequency 40 MHz; focal length 6 mm; depth of penetration 5–15 mm; 30–40 µm axial and 70–90 µm lateral resolution). The time gain compensation and other operating parameters were kept constant throughout experiments (frequency = 40 MHz, frame rate = 16 images/s, gain = 30 dB, depth = 11 mm, width = 13.00 mm, dynamic range = 60 dB, sensitivity = high; transmit power = 100%) to avoid experimental bias. Each liver HFUS acquisition, including overall animal preparation, took no more than 15 min. During recovery, all animals were monitored for any signs of pain or discomfort.

#### 2.4.2. CUS Scoring

B-mode cine-loops were analyzed offline by two experienced veterinary radiologists (S.G., M.G.) who collaborated to reach a consensus on the interpretation of US features. For each mouse, at the chosen time points, HFUS images were selected that represented the best frames in the cine loops for the liver lobes of interest, avoiding motion-related artifacts (in the expiratory and diastolic phases based on the recorded electrocardiogram/respiratory tracings) as well as bone- or intestinal gas-related shadowing. Since histopathological scores are based on evaluation of lipid-droplets accumulation in hepatocytes, we first examined traditional scattering parameters from the echo amplitude histogram of normal and steatotic liver parenchyma. Briefly, the presence and grading of liver steatosis were assessed by visual interpretation of HFUS features, based on protocols validated in both patients and mouse models, expressed using the semiquantitative CUS scoring system: (0) homogeneous liver parenchyma of medium level echogenicity; (1) diffusely increased parenchymal echogenicity; (2) discrete coarsened and heterogeneous parenchymal echogenicity; (3) extensive coarse and heterogeneous parenchymal echogenicity [15,16]. Furthermore, the visual appearance of liver echogenicity was rated relative to the renal cortex as (0) lower or (1) equal or (2) higher, as well as the absence (0) or presence (1) of ascites [18].

#### 2.4.3. QUS Parametric and CAD Texture Analysis

Echogenicity and heterogeneity were also numerically measured by calculating QUS indices. The hepatic–renal ratio (HRI) was easily determined with the standard tools of the scanner’s proprietary software (Vevo Lab software version 3.0.0). HFUS images were analyzed offline by the same radiologists (S.G., M.G.) in consensus, who collaborated to agree on the correct selection of images and placement of ROIs on anatomical regions based on established technical criteria. HRI was calculated by normalizing the caudate lobe echogenicity to that of the right renal cortex, potentially highlighting the fat-induced increase in hepatic brightness on gray-level histogram (GLH) [29,30,31,32]. Theoretically, this ratio can compensate for variability related to scanning parameters and tissue attenuation and has shown moderate correlation with liver biopsy in humans [33,34,35]. The methodology used has been explained in depth elsewhere [18]. Furthermore, the diagnostic performance of HFUS could be improved by extracting additional texture features. In the literature, several CAD algorithms have been proposed for feature extraction and selection from medical images of steatotic livers, from simpler intensity features to more sophisticated ones. In particular, the radiomic features commonly used to estimate the granularity and heterogeneity of diffuse liver diseases include the second-order textural parameters (SOP) named Gray-Level Co-occurrence Matrix (GLCM), that provide information on the spatial relationship of intensity values in adjacent pixels [10]. Considering that a combination of texture features can improve the accuracy of steatosis detection, the smallest set was chosen to provide useful integrative information, based on the relevant literature on liver US or extended from other types of US radiomic studies [7,24,36,37]:

− Sum Entropy: Measures the randomness in the texture of the image; a higher value indicates greater variation in the pixel intensity across the image, indicating a more heterogeneous texture.
− Energy or Angular Second Moment: Measures the homogeneity in the texture of the image; a higher value indicates greater uniformity; conversely, a more complex texture.
− Contrast: Measures the local variations in the image; a higher value indicates greater intensity differences between neighbor pixels; conversely, the contrast drops to zero.
− Correlation: Measures the linear dependency between pixel pairs in the image; a higher value indicates a more constant texture.

**Figure 2:**
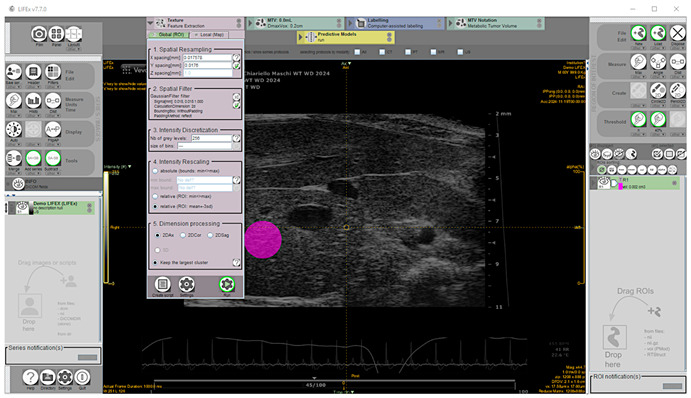
Representative screenshot of the texture parameters measurement workspace for the selected region of interest shaded in pink.

These SOP features have the advantage of taking into account both total liver lipid content and fat deposit distribution [38] and are not dependent on the pixel intensity in the image and image acquisition settings [39]. A total of 135 images, including 87 from mice diagnosed for hepatic steatosis of any degree on histology, and 48 from mice for which the disease was excluded, were analyzed offline. The texture feature extraction from HFUS images was performed using the open-source application LIFEx v7.7.0 (LITO, Curie, Iserm, CNRS, Paris-Sud, University Paris Saclay, Orsay. Download available from 18 November 2024) [40]. Proprietary file formats were converted to the Digital Imaging and Communications in Medicine (DICOM) format and stored in a digital image archive. The workflow of the proposed CAD scheme consisted of the following steps: (1) image selection; (2) liver segmentation; (3) image pre-processing; (4) feature extraction; (5) feature selection. Briefly, a complete set of HFUS images in DICOM format was loaded into the Graphical User Interface (GUI), then one HFUS image was selected for each scanned liver lobe at a defined time point, representing the best frame in the cine loop (according to the criteria described above). To perform segmentation, a single-stack image series must be saved from RGB color recorded layers as active reference using the “save series” tool (format: DICOM; size: 2D) and the chosen frame loaded. To ensure consistency and reliable statistics, a 100-voxel circular region of interest (ROI) was manually placed on the liver parenchyma in the focal area as close as possible to the image center to minimize echo distortion artifacts, avoiding vascular or biliary system structures. [41]. Subsequently, a low pass 2D Gaussian filter was applied to the ROI images for speckle noise reduction and feature repeatability improvement (kernel: X, Y = 0.018 mm; padding method: reflect, on the exact bounding box of the activated ROI without additional padding) [14,42,43]. Before texture analysis, signal intensity in the ROI was corrected by the mean and three standard deviations to minimize overall grayscale variation between images and scanners [41]. Then, texture-related features were computed (spatial resampling: x-spacing = 0.017578 mm, y-spacing = 0.0176 mm; intensity discretization ROI drawn: N. Gray Level = 256; bin size = 0.30; intensity rescaling: relative) [43,44] (Figure 2).

Overall, CAD scheme computed image features and created three feature pools for each experimental group, based on observational ages (8, 16, 24 weeks). Each CAD analysis on HFUS, including total liver lobes from an animal to ensure a representative sampling of the liver parenchyma, took no more than 15 min.

### 2.5. Histological Examination

The liver specimens were collected 1-day after the last HFUS examination to ensure the validity of the histology reference standard, due to the variation in hepatic fat contents over time [45]. After sacrifice, liver was collected from each mouse and formalin-fixed according to standard procedures. Thereafter, 7 μm sections were cut from each paraffin block and stained with hematoxylin and eosin (H&E) for morphological evaluation. In order to evaluate the relationship between CUS score and histologically determined steatosis, an experienced pathologist (VB) classified the severity of liver steatosis in each mouse based on a validated preclinical scoring system, adapted to histological peculiarities of rodent’s species [46,47] from the human NASH–Clinical Research Network scoring system [48,49,50]. Given the different ways of reporting disease scores in the literature, we have provided a detailed description of the specific approach used to measure the hepatic steatosis severity score for each mouse, as recommended [51].

In particular, steatosis was determined by analyzing the following histological features (5 fields per animal, 200× agnification):

− Steatosis: Percentage of the total area occupied by hepatocytes showing microvesicular (multiple lipid droplets inside the hepatocytes) and macrovesicular (a big lipid droplet displacing the nucleus) alterations as 0 (absent); 1 (mild, <10%); 2 (moderate, 10–30%); 3 (severe, >30%).
− Hypertrophy: Percentage of the total area occupied by hypertrophic hepatocytes (cellular diameter > 1.5 of a normal hepatocyte) as 0 (absent); 1 (<10%); 2 (10–30%); 3 (>30%).

The final steatosis severity score (SSS) was determined by the sum of the semiquantitative scores for macrovesicular and microvesicular steatosis separately, and hypertrophy. A Nikon Eclipse E600 light microscope equipped with a digital camera and the Nis element v3AR software was used (Nikon Instruments, Melville, NY, USA). For each animal, at least three sections were examined, including distinct liver lobes, avoiding microscopic fields in which hepatic venules and portal tracts were visible.

The pathologist also assessed inflammation and fibrosis as additional features of MASLD according to the above-mentioned scoring system (3-point scale for classifying mild, moderate, and severe changes).

The radiologist and pathologist were blinded to each other’s imaging and histology scores, as well as biochemical data at the time of endpoint.

### 2.6. Statistical Analysis

In this experimental protocol, we examined the effects of SD and WD in C57BL/6J WT mice and a GEMM with C57BL/6J genetic background for comparison. The experimental group included 7 WT and KO SD-fed mice (*n* = 7) and 15–16 WT and KO WD-fed mice (*n* = 15–16). The sample size of the experimental groups was calculated taking into account the ethical recommendation to reduce the number of animals as much as possible and corrected for 10% expected attrition [52,53]. Means of the expected data extrapolated from the relevant scientific literature were compared [54] to obtain statistically significant results for differences of at least 30% (Reference mean = 100; Test mean = 70) between the average parameter values (http://www.biomath.info/power/ttest.htm, (accessed on 1 September 2020) because project authorization of Italian Ministry of Health was March 2021.) [55]. In the end, 80% power, 20% standard deviation, and a *p* ≤ 0.05 were used. An exploratory assessment of metabolic changes was performed to translationally guide further analysis of imaging data. The experimental data were processed using descriptive statistics. Statistical analyses were performed using GraphPad Prism software version 8.0 (GraphPad Software, Inc., San Diego, CA, USA). The screening value of CUS staging and QUS parameters was evaluated in terms of the following metrics: sensitivity, specificity, accuracy, and positive predictive value. Box-and-whisker plots were created for exploratory comparison of datasets distribution. Spearman’s rank coefficient was used to investigate the correlation between the US parameters and the histology steatosis grade, expressed as r. Correlation coefficients were interpreted as described elsewhere [14]. *p* values < 0.00833 were considered significant in all analyses by applying the Bonferroni correction to adjust for multiple statistical tests.

## 3. Results

### 3.1. Main Phenotypic Characteristics

Biometric, nutritional, and blood biochemistry data were summarized to suggest preliminary hypotheses for more complex analysis (Table 1 and Table 2). Indeed, overweight consequent to unbalanced nutritional conditions leads to the infiltration of fat in several organs including the liver, especially if it is associated with insulin resistance and dyslipidemia [56]. In the clinical setting, the presence of comparable risk factors suggests the opportunity to monitor hepatic steatosis in patients, aiming for early diagnosis and potentially more effective treatments to prevent or slow the progression of liver disease. In particular, liver echography is often the first-line diagnostic tool for steatosis, because it is relatively non-invasive, readily available, and cost-effective compared to other methods. In this context, we explored the effects of WD on WT and KO mice on metabolic parameters.

WT and KO mice showed differential body weight and feeding responses to WD. Specifically, WT mice fed WD increased their body weight by 25.65% compared to WT SD mice, while KO mice fed WD increased their body weight by 47.39% compared to KO SD mice, with changes in body weight nearly 85% greater than WT mice. Similarly, WT mice fed WD increased their caloric intake by 9.8% compared to WT SD mice, while KO mice fed WD increased their caloric intake by 17.5% compared to KO SD mice, showing 79% greater energy consumption than WT mice when given access to WD. Indeed, WT mice fed WD increased their feed efficiency by 20% compared to WT SD mice, while KO mice showed a 60% increase for this parameter under WD, a three-fold increase compared to WT mice. No group of mice showed mean values of serum parameters suggestive of impaired renal function (BUN: 34–58 mg/dL; creatinine: 0.3–1.1 mg/dL) [57]. By monitoring changes in non-fasting cholesterol, triglycerides, and glucose from 8 (baseline, pre-diet) to 16 weeks of age, we were able to uncover subtle and early changes in metabolic homeostasis (Table 2). Indeed, it has been reported that postprandial glycemia is more sensitive to the early stages of diabetes and that non-fasting lipid values change minimally compared to fasting ones and are correlated with cardiovascular risk in metabolic syndrome [58,59].

As expected, WD affected both lipid and glucose metabolism. In particular, blood cholesterol increased by approximately 30% in WT and KO mice fed WD, while triglycerides unexpectedly decreased by approximately 60% in the same feeding conditions (Table 2). This finding has been described in other studies in C57BL/6J mice fed a high-fat, high-cholesterol diet and explained by reduced synthesis and/or increased clearance of triglycerides, or by reduced hepatic Very Low-Density Lipoprotein (VLDL) secretion/increased triglyceride uptake from adipose tissue due to acute insulin elevation [60,61]. This global trend was observable also at the experimental endpoint (24 weeks of age), with consistent alterations in fasted serum cholesterol, triglycerides, glucose, and insulin in WD-fed mice of both genotypes compared to SD-fed mice, as well as increased levels of alanine aminotransferase (ALT) and aspartate transaminase (AST), further suggesting liver damage with an unbalanced diet, as described in detail elsewhere [18,25]. Since the objective of this study was the analysis of selected US features, a detailed elaboration of the phenotypic metrics would be beyond the scope of this article [25].

Taken together, these data suggested that the C57BL/6J mouse model of WD-induced obesity shows several similarities to the human metabolic syndrome, including overweight, insulin resistance, dyslipidemia, and hepatic transaminases alterations, which may be associated with hepatic steatosis. Modifications on this genetic background, such as the deletion of the MAPK15-ERK8 gene, could impact this pathological phenotype. Therefore, we evaluated the translational utility of in vivo HFUS monitoring of liver changes in these mouse models related to dietary and/or genetic interventions by testing an integrated USICA approach.

### 3.2. Comparison of In Vivo CUS Determinations with Histological Features on Liver Samples

Based on histological criteria examined in the 45 mice studied, predominantly macrovesicular (area%, 4.55 ± 5.54) or microvesicular (area%, 19.01 ± 21.82) steatosis of any extent were observed in approximately 9% and 13% of mice, respectively, whereas a mixed pattern (area%, 36.08 ± 24.17) of small and large lipid droplets of any extent was seen in 78% of mice, including hypertrophic hepatocytes in 31% of mice. HFUS changes suggestive of hepatic steatosis were observed in 36 (80%) mice.

**Figure 3:**
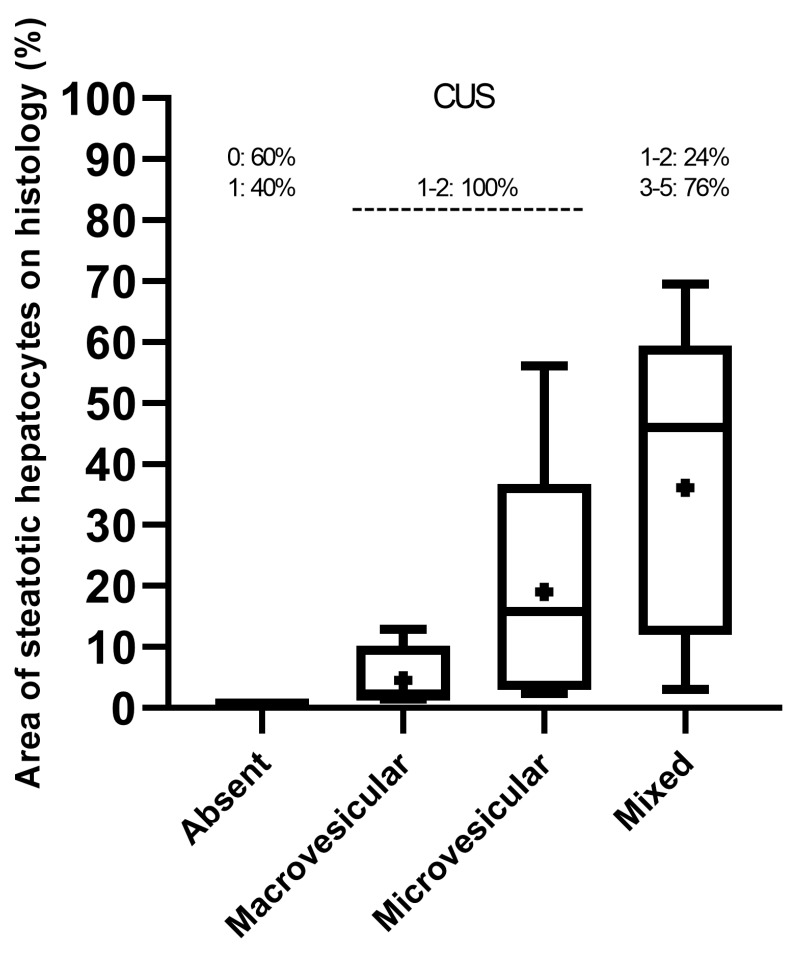
Distribution (median, mean, and range) of histological criteria sorted by CUS scoring.

Overall, median, mean, and interquartile ranges (IQR) of the area of steatotic hepatocytes on histology increased progressively from the mice groups that predominantly presented macrovesicular or microvesicular steatosis to those showing more complex “mixed” histological features (macrovesicular plus microvesicular steatosis) (Figure 3). However, the distribution of the “mixed steatosis” group appeared negatively skewed, showing a higher frequency of low values, in contrast to those of predominantly macrovesicular or microvesicular steatosis, which were positively skewed.

All mice exhibiting predominantly macrovesicular or microvesicular steatosis showed the lowest CUS scores (1–2), while mice with mixed histological pattern mainly showed higher CUS scores (3–5) (Figure 3).

The prevalence of the histological scores for the diagnosis of hepatic steatosis of any severity in experimental groups at 24 weeks of age is shown in Table 3. Detailed information on the key features analyzed by the ultrasonographic and histological scoring systems are described in the Supplementary Materials (.xlsx file: S1_SSS_CUS.xlsx). Overall, CUS showed good screening performance in monitoring the progression of hepatic steatosis severity in the same subject over time. By comparing ultrasonographic features of hepatic parenchyma at 24 weeks of age with the histology as reference truth, CUS successfully detected 100% (sensitivity) of steatosis in mice (True Positive Rate, TPR = 1), with a specificity of 60% (True Negative Rate, TNR = 0.6), an accuracy (number of correctly classified subjects) of 73%, and a Positive Predictive Value (PPV) of 83%. In particular, the sensitivity of HFUS progressively increased from 60% for CUS = 2; to 70% for CUS = 3; to 90% for CUS = 4; and to 100% for CUS = 5, demonstrating the ability to reliably detect the early stages of disease when the total area of steatosis exceeded at least 10% and improving the accuracy for moderate to severe steatosis (fat infiltration of 30% or more). Overall, CUS had a very strong positive correlation with histological grading (Spearman r = 0.8598, CI= 0.7536–0.9233, *p* < 0.0001).

**Figure 4:**
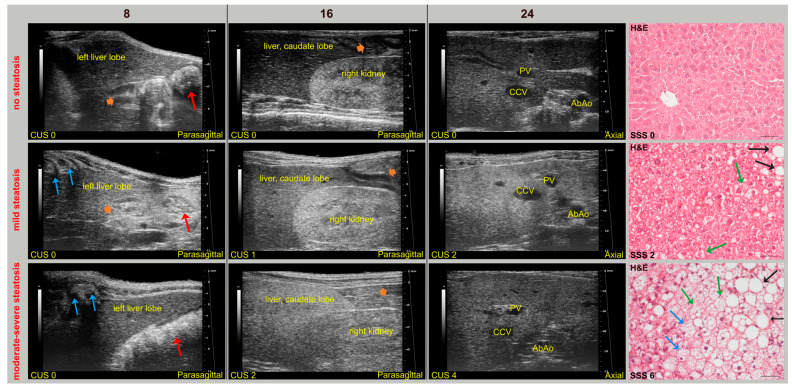
Selected HFUS images from longitudinal studies (8, 16, 24 weeks of age) in mice, representing CUS in a subject without steatosis (WT, SD) with mild (WT, WD) or with moderate–severe (KO, WD) steatosis (from upper to lower row) at histology (H&E, Scale bar 50 μm). (**Top** row): HFUS appearance of the liver in a mouse without steatosis (CUS 0, SSS 0). The liver parenchyma has a homogeneous echostructure and is less echogenic than the right renal cortex, in a constant manner over time. The corresponding histological image shows hepatocytes with eosinophilic cytoplasm and centrally located nuclei. (**Middle** row): HFUS appearance of the liver in a mouse with mild steatosis (CUS 2, SSS 2). Parenchymal echogenicity showed a progressive and widespread increase over time compared to that observed at 8 weeks of age, with echogenicity of the caudate lobe equal to that of the renal cortex as early as 16 weeks of age. Corresponding histological image shows mainly macrovesicular (black arrows) steatosis and some signs of microvesicular (green arrows) steatosis. (**Bottom** row): liver HFUS appearance in a mouse with moderate–severe steatosis (CUS 4, SSS 6). The liver parenchyma showed a progressive increase in echogenicity and a coarsened texture over time compared to that observed at 8 weeks of age, with the echogenicity of the caudate lobe progressively going from being equal (16 weeks of age) to greater (24 weeks of age) than that of the right renal cortex. The corresponding histological image shows, in addition to macrovesicular (black arrows) and microvesicular (green arrows) steatosis, hepatocytes degeneration characterized by enlarged size, with the nucleus often peripherally located and clear cytoplasm (blue arrows). Red arrows: stomach; orange arrowheads: mucous or gaseous pattern in bowel, with related acoustic shadowing artifacts; blue arrows: ribs with associated acoustic shadow.

Overall, SD-fed mice had a homogeneous and isoechoic liver parenchyma throughout the study, whereas the liver parenchyma of WD-fed mice tended to become progressively hyperechoic and heterogeneous, with hepatic echogenicity equal to or greater than that of the renal cortex. No mice showed ascites. In addition, mild inflammation and fibrosis were observed in the liver of WD-fed mice (62.5% and 25% of WT mice, 87% and 80% KO mice, respectively). Interestingly, a WT mouse and a KO mouse fed SD showed mild sonographic parenchymal heterogeneity, although histological features of steatosis were absent. Nevertheless, evident glycogen content in hepatocytes or mild fibrosis, with different distribution in liver lobes, were observed in these mice by Periodic acid–Schiff staining and Masson’s trichrome, respectively. Similar findings were also found in four WT WD-fed mice (False Positive Rate, FPR = 0.4). These specificity issues could be explained by considering that fat, glycogen, and fibrotic tissue can cause increased HFUS scattering, producing similar sonographic changes in liver alterations other than steatosis [62,63]. Indeed, mild-to-moderate hepatic steatosis was confirmed by histology in three SD-fed KO mice (43%), which exhibited corresponding changes in US features over time. In particular, histology-confirmed US findings of steatosis started to appear at 16 weeks of age in both WT (62.5%) and KO (100%) mice fed WD, with overall higher severity in KO mice. Additionally, HFUS images were examined to detect renal abnormalities. In agreement with biochemical tests, no mouse showed an abnormal US renal appearance, with clear visualization of the corticomedullary border, renal papilla, and echogenic renal capsule. Representative HFUS liver views and related histological images are provided in Figure 4.

Taken together, these results demonstrated that CUS is a noninvasive and readily available imaging technique that allows longitudinal monitoring of hepatic steatosis in murine models in a translational manner and from an early stage. Furthermore, we were able to highlight phenotypic differences in murine liver related to genetic and/or dietary factors. Finally, by avoiding sacrifice at multiple time points, HFUS supported more ethical preclinical research.

### 3.3. Comparison of In Vivo QUS Determinations with Histological Features on Liver Samples

Detailed information about QUS and histological scores of the mice are presented in Table 4.

We explored the relationship between the QUS findings and the histopathological quantification of MASLD. Using HFUS parameters computed from 24 weeks of age, HRI had a strong positive correlation with histological grading (Spearman r = 0.6893, CI = 0.4894–0.8204, *p* < 0.0001). The mean HRI was 0.691 ± 0.082 (0.492–0.803) for no steatosis, 0.809 ± 0.149 (0.621–1.094) for mild steatosis (histological score 1–2), 1.003 ± 0.128 (0.771–1.153) for moderate steatosis (histological score 3–4), and 1.110 ± 0.28 (0.584–1.621) for severe steatosis (histological score 5–7). Choosing the cutoff of 0.803 (i.e., the maximum value found among subjects with SSS = 0) as the HRI value to discriminate between healthy subjects and subjects affected by steatosis, the HRI showed a sensitivity of 70%, an accuracy of 80%, and a specificity and a PPV of 100%.

**Figure 5:**
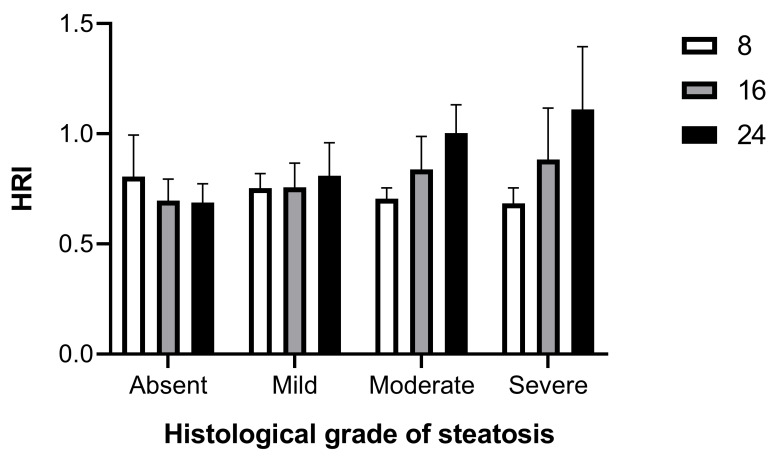
Changes in HRI from baseline age (8 weeks) over time (16, 24 weeks) by degree of histological steatosis.

In fact, the HRI between 0.622 and 0.803 represents an indeterminate range that includes both healthy mice and some mice with mild (15.5%) and moderate–severe (4.5%) hepatic steatosis. Based on pathological correlation, 80% (36/45) of HRI measures had a PPV of 100% for predicting steatosis. Furthermore, the usefulness of HRI to discriminate the severity degrees of steatosis was evaluated against histologic results. Setting a cutoff of 1.095 (i.e., the maximum value found in subject showing SSS = 1–2), the HRI showed a sensitivity of 47%, an accuracy of 70%, and a specificity and a PPV of 100% to discriminate between subjects with mild and moderate–severe hepatic steatosis. Interestingly, when longitudinally monitored HRI values were sorted by histological grade of steatosis confirmed at 24 weeks of age, a trend of progressive increase in HRI over time could be observed in subjects with mild hepatic steatosis, which became clearly evident in those with moderate and severe liver disease (Figure 5).

Overall, these results demonstrated that HRI can be an effective imaging tool for noninvasive screening mouse models for the diagnosis of hepatic steatosis and, although less efficiently, for classifying the severity of hepatic steatosis.

### 3.4. Comparison of In Vivo CAD Texture Analysis with Histological Features on Liver Samples

**Figure 6:**
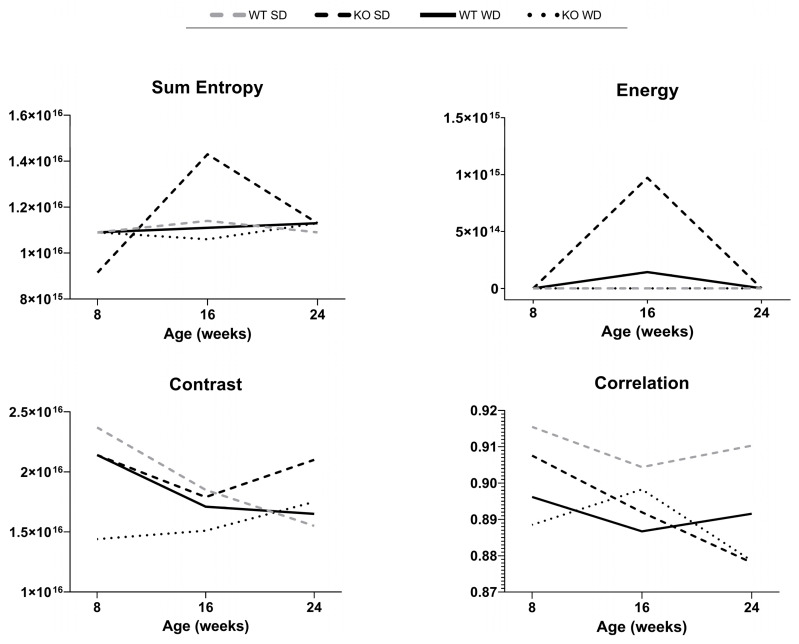
Graphs representing the distribution of mean values for the selected set of texture features across experimental time points (8, 16, and 24 weeks of age) in WT and KO mice fed SD or WD.

Texture features were successfully measured for all mice and time points. The values of each texture feature computed were averaged over the images of all liver lobes analyzed for each animal at each age (Figure 6).

Although GLCM texture features indicative of echogenicity homogeneity (contrast and correlation) showed greater variability than those suggesting a more heterogeneous and complex echostructure (sum of entropy and energy), no clear hypothesis can be postulated from visual analysis of their distribution patterns and descriptive statistics over time across experimental groups. Analysis by dividing subjects into experimental groups resulted uninformative probably because hepatic steatosis exists on a spectrum and there were mice with mild steatosis in the SD-fed KO group, as well as mice without hepatic steatosis in the WD-fed WT group, with overlapping characteristics among subjects of different groups. Interestingly, the WT SD group theoretically included all healthy subjects, as confirmed by histological scores. This group showed no obvious changes in total entropy, energy, and correlation over time, while contrast showed a decreasing trend during the process of physiological aging (−40%), suggesting that GLCM contrast could be a useful parameter in detection of small, age- or disease-related changes in liver texture not visible with conventional HFUS.

**Figure 7:**
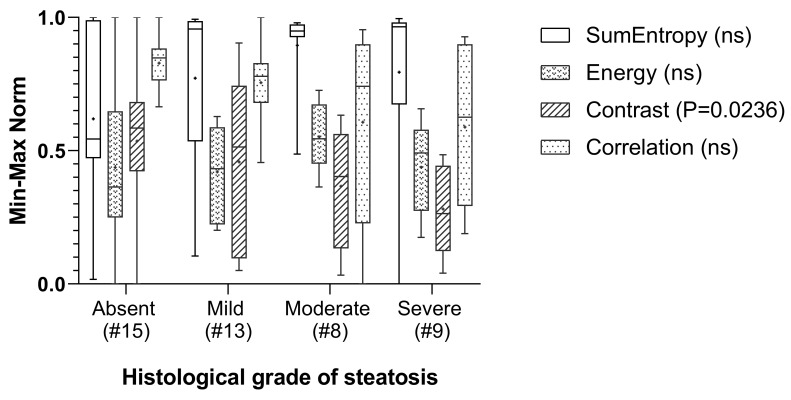
Distribution (median, mean, and range) of CAD texture features, Min-Max normalized and sorted by histological severity of hepatic steatosis in 24-week-old WT and KO mice fed SD or WD.

Thus, such explorative findings pointed out that grouping might over-simplify the analysis, whereas a more detailed, individual-based analysis, considering the degree of steatosis in each subject, may reveal more informative patterns and associations. A useful approach for exploratory studies is regression models, which assess how continuous variables, such as the degree of steatosis, influence outcomes rather than groups. More meaningful results were suggested by the analysis of the distribution of GLCM features normalized to the minimum–maximum range (0, 1) and classified into the categories of absent or present steatosis according to the histological reference, and are shown in Figure 7.

No statistically significant correlations were identified between the selected GLCM texture features and histological grading. Although in our explorative analysis the textural parameter contrast had a moderate negative correlation with the histological score (Spearman r = −0.3369, CI = −0.5797–−0.03918, *p* = 0.0236), the corresponding *p* value was not statistically significant with the applied Bonferroni correction. Nonetheless, in the discrimination analysis, the median and mean SSS values for the contrast parameter were the highest in healthy subjects and showed a downward trend from mild to severe steatosis. It is known that the increase in lipid droplets and the consequent displacement of nuclei and organelles within hepatocytes, during the progression of hepatic steatosis, can influence ultrasound scattering and attenuation, reducing local contrast intensity. In our dataset, the downward trend in median and mean GLCM contrast values with advancing steatosis suggests a reduction in intensity differences between adjacent pixels as well as potentially increased echogenicity, which may be biologically correlated with increased fat deposition in more severe stages [24]. Comparably, the range of GLCM correlation values became progressively wider, with an upward-shifted distribution and a reduction in the median and mean, overall indicative of greater variability in the gray level and a higher frequency of low values when moving from healthy mice to those with mild and moderate-to-severe steatosis. This finding suggests a greater complexity in the structural pattern with worsening steatosis in the mouse models examined [3]. Less noticeably, the sum of entropy and energy showed a narrowing of the range and an increase in the median and mean values when comparing the non-steatosis group with those with mild and moderate–severe steatosis, reflecting a trend toward an irregular distribution of echointensity within the liver tissue, probably related to a patchy fat infiltration in hepatocytes. Overall, the results of our pilot study failed to clearly demonstrate that US texture analysis, as a complement to CUS and QUS, would significantly improve the discrimination of hepatic steatosis in mouse models, at least in the early stages of diseases. Nevertheless, they highlighted the potential for further research to explore the emerging issue of the non-invasive imaging classification of steatosis through automated AI-based US analysis in preclinical models, in line with the current scientific literature.

## 4. Discussion

Metabolic syndrome and overweight are associated with an increased incidence of MASLD worldwide. When MASLD is diagnosed early, before significant inflammation or fibrosis develops, it is easier to treat or reverse.

US imaging is commonly used in clinical settings to diagnose and monitor MASLD because it is safe, widely available, and less expensive than other imaging modalities.

The availability of mouse models resembling this condition, in which liver steatosis could be assessed without necropsy and monitored over time from early to advanced stages, would increase the efficiency of translational research in theranostics.

Longitudinal HFUS imaging has proven useful for phenotyping rodent models of hepatic pathology including MASLD [15,17,18,21,29,30,31], both WT and GEMMs [64]. During MASLD, HFUS allows us to monitor the pathological changes in the liver and other involved organs in the same animal over time, evaluating the effects of dietary or pharmacological treatments, as well as genetic modifications, and highlighting the individual variability within the group. From a scientific and animal welfare perspective, HFUS can be beneficially used to evaluate mouse models, representing a translational tool that minimizes animal suffering. Nowadays, HFUS devices and ancillary equipment dedicated to laboratory rodents are widely available, although their correct use requires specialized expertise. In this pilot study, we evaluated the utility of HFUS in predicting the detection and severity of hepatic steatosis in vivo in two murine genotypes, C57BL/6J WT and MAPK15-ERK8 KO mice, with the same genetic background, fed SD or WD. The observations made in our mouse models are representative of the majority of prospective preclinical studies evaluating the role of HFUS in murine models with histologically confirmed hepatic steatosis. Overall, they demonstrate the value of this imaging technique for the non-invasive diagnosis and monitoring of liver pathologies in laboratory rodents. In particular, we compared CUS with USICA for predicting histology-confirmed steatosis in adult mice with MASLD. Furthermore, data obtained from SD-fed WT C57BL/6J mice may further contribute to reference databases of normal murine liver. Serial HFUS examination has allowed us to characterize various severity degrees of hepatic steatosis and monitor their progression in the same animal, with CUS strongly correlated with the grade of steatosis on liver histology. This finding is consistent with human data, in which CUS accurately predicts the histological classification of hepatic steatosis in at least 50% of cases, compared with approximately 75% using the more complex MRI [45]. Given the significant public health impact, early diagnosis of MASLD is important to timely test interventions aimed at reversing or reducing hepatic steatosis. Simple steatosis is the initial alteration in the evolution of MASLD, and we were able to detect it by HFUS in our mouse models as early as 4 months of age and at the mild clinical stage (<10%), subsequently confirming its association with microscopic alterations. From a preclinical perspective, HFUS may be useful to determine the optimal experimental timing for testing new treatments or for sacrificing animals to characterize the response to therapy at a given stage of liver disease, despite interindividual variability. In this regard, we have demonstrated that HFUS has the ability to examine mouse models that more closely reproduce the early and mild stages of fatty liver disease caused in humans by unbalanced WD, with relevant translational implications. As critical concern, two SD-fed mice (one for each genotype), and four WD-fed WT mice showed a mild increase in echogenicity and in heterogeneity of the liver parenchyma at 24 weeks of age, while histology evidenced glycogen storage or fibrosis in the absence of steatosis. These findings are in line with the literature, and likely relate to comparable US scattering produced by fat, glycogen, and fibrotic tissue [15,62,63]. Despite a relatively low specificity, CUS demonstrated the highest correlation coefficient with histological grade of steatosis. This result could probably be related to the competencies of the radiologists involved, underlining the importance of expertise in the specific field. Indeed, for an accurate qualitative interpretation, the HFUS images were analyzed by two experienced veterinary sonographers and, once an agreement was reached, the results were considered reliable. A critical issue is that the relative ease of acquisition of HFUS images by non-specialized researchers compared to other imaging modalities may result in a lack of rigor in the interpretation of imaging results. Furthermore, it is important to examine the different lobes of the liver, because variations in the severity of steatosis have been observed in different liver zones in animal models, consistent with the literature [64]. Our results also demonstrated that quantification of hepatic steatosis by HFUS is feasible in murine models of MASLD. Although adequate expertise is also required to properly use HFUS equipment and define image acquisition and analysis protocols, automatic feature detection and quantification using AI could help reduce the operator dependency on US image interpretation. Therefore, we hypothesized that integrating multiple objective texture features computed from HFUS images could be advantageous compared to using the subjective internal echoes analysis alone. We proposed simple descriptors with diagnostic capability comparable to that of more complex models, computed using proprietary US device tools or by open-source GUI. Clinical observations indicated that QUS might be more objective than CUS for grading hepatic steatosis in patients [43]. However, accurate assessment of quantitative parameters, such as HRI, requires experience and knowledge of the technical requirements of diagnostic US, both in image production and interpretation.

Currently, QUS cannot completely replace clinical experience even in the preclinical field, whereas quantitative data should rather be integrated with clinical and CUS results. Furthermore, technical knowledge of both US physics and anatomical landmarks, as well as optimization, standardization, and detailed description of methodologies are of paramount importance to ensure accurate results. Hayakawa and colleagues assessed hepatic steatosis in 19-week-old C57BL/6J male mice, fed a choline-deficient, high-fat diet and assuming dextran sulfate sodium, by determining the ultrasonographic liver to kidney brightness ratio, and highlighted a significant correlation with histological findings (Spearman’s rank correlation coefficient 0.3583, *p* = 0.0183) [65]. In our study, we found a strong positive correlation between HRI and SSS (r = 0.6893, *p* < 0.0001). Although we used a WD that leads to simple steatosis in mice, our improved result may be explained by the use of a dedicated HFUS scanner for laboratory rodents equipped with high-resolution probes. Nevertheless, in agreement with the literature, the sensitivity for detecting steatosis using HRI appeared higher for moderate and severe grades of steatosis and lower for mild steatosis [66]. Interestingly, three KO mice with higher SSS (5, 7) showed lower HRI values than other mice with comparable degree of steatosis. Similarly to the findings in human patients, HRI values were very close for higher degrees of steatosis severity. These findings could be explained by the interaction between an increased attenuation of US, proportional to the intracellular accumulation of fat vacuoles, and the variability of reflective interfaces related to the prevalent pattern of steatosis, as well as by the heterogeneous involvement of the liver lobes [66]. Indeed, the WD chosen for this study caused both macrovesicular and microvesicular steatosis. The factors that lead to these two types of alterations are still poorly understood, as is which form might cause less or more liver damage [67]. Macrovesicular or microvesicular patterns of hepatic steatosis have been found in both humans and murine models of MASLD [46,68]. However, the sonographic effect of each form of intracellular fat deposition in the liver parenchyma has not been fully characterized [69]. Based on the results of the literature survey, we selected a limited set of texture features to quantify the GLH pattern changes in the liver, complementing MASLD monitoring. Indeed, in addition to increased echogenicity, the progressive accumulation of fat droplets in hepatocytes can lead to a more complex echostructure with greater heterogeneity, due to the variable reflection of ultrasound waves by multiple and irregular interfaces. The potential of radiomics analysis to gain a deeper understanding of the complex interplay of liver imaging phenotype, better correlating it with histopathology, has been explored primarily in the clinical setting using MRI-extracted features from tumor lesions or fibrotic livers, as well as in rodent models [70,71]. AI-based applications in US techniques have great potential in translational research, but they are still in their early stages and have some challenges to overcome, such as limited sample sizes in preclinical studies, standardization of methods, and pre- and post-processing of data [23,72]. Although high noise level is an inherent challenge in US, its practical role in noninvasive diagnostic screening of liver diseases has prompted significant efforts to extract texture features from US images, and GLCM has been selected as being promising in distinguishing the spatial distribution and pattern of pixel intensities [23,24,71]. GLCM features show the frequency with which different combinations of gray levels occur in an image and are widely used to study the relationship between neighboring pixels, particularly in liver disease research. However, results presented in some preclinical studies would not be directly translatable to the in vivo setting, because the use of the ex vivo liver specimens could introduce biases, such as tissue changes related to ischemia due to animal sacrifice or sample fixation [24,71].

Despite the promising results of the data analysis for CUS and HRI, the small sample size of this preliminary study poses limits for the optimal selection of texture features by using classifier algorithms on a large pool [73,74]. Overall, the literature highlights that this topic has not yet been widely studied and requires further investigation, also in relation to imaging techniques with more advanced image quality, like MRI or CT. Effective development of preclinical CAD schemes from US images would likely benefit from a larger dataset, as at least one of the four correlations in our pilot study showed a moderate effect size of >0.30, but the corresponding *p* value was not statistically significant. Nonetheless, adhering to the 3Rs, this study deliberately sought to minimize animal suffering and demonstrated the feasibility of extracting HFUS texture features in mice from early to advanced stages of hepatic steatosis. The different distributions and descriptive statistics of GLCM features explored in our dataset are likely explained by the changes in US reflectance due to the physical properties of fat. From a biological point of view, increased uniformity of echo intensity, reflected by lower contrast, as well as a more irregular distribution of the echo pattern, suggested by increased entropy and energy, are indicators of worsening hepatic steatosis, since fat accumulation in liver cells disrupts the normal microstructural organization. The relationship between contrast and steatosis might differ depending on the severity of the condition. While there is a general tendency for GLCM contrast to decrease over time in liver steatosis due to increased tissue homogeneity, the exact trend can be influenced by factors like the stage of steatosis, the presence of fibrosis, and the imaging technique used. Additionally, increasing brightness can affect the texture of the liver, making it more uniform or showing a coarser fat distribution [75,76].

We believe that our preliminary results, showing significant correlations between HFUS and histological findings, suggest a consistent effect size for some imaging assessments and that the proposed CAD approach has the potential to guide future studies on the use of US texture features to detect early structural changes in murine steatotic livers. Further research in this area could contribute to the development of better CAD workflows suitable for HFUS texture analysis. Interdisciplinary collaboration between radiologists and medical physicists or engineers, combining expertise in anatomy, physiology, pathology, and animal model management with signal processing capabilities, can foster the development of algorithms enabling the reliable extraction of texture features from US images in the preclinical setting. Finally, HFUS was able to highlight differences in liver disease progression related to genetic and/or dietary factors, from early to advanced stages, without the need to sacrifice the animals at multiple time points, thus more faithfully reproducing what occurs in the clinical setting in patients with suspected hepatic steatosis. As shown in Table 3 and Table 4, consistently across US and histology, we were able to detect by imaging an overall higher incidence of hepatic steatosis in KO mice compared to WT mice, already finding few cases in the SD-fed KO group (3/7, 43%). As expected, the incidence of hepatic steatosis differed between SD-fed and WD-fed WT mice. In addition, we observed a greater number of steatotic mice in the WD-fed KO group compared to WD-fed WT mice (15/15, 100% versus 12/16, 75%, respectively), as well as higher values of both ultrasonographic and histological severity scores with comparable frequency distribution.

The novelty of this exploratory study refers to the genetic peculiarity of the included sample sets and the integrated analytical workflow, combining qualitative and parametric HFUS analyses in both wild-type and MAPK15-ERK8 knockout mouse models fed a WD to detect longitudinal changes in liver. The use of objective quantitative texture features with the qualitative pattern of US echoes can contribute to the accuracy of steatosis detection and monitoring in preclinical research, especially if parameters with discrimination performance comparable to more complex ones, e.g., features extracted from GLH, can be directly calculated using HFUS device tools or open-source programs. Furthermore, to the best of our knowledge, this study is the first to determine the US texture characteristics of steatotic liver in both living WT mice and GEMM mice by CAD.

The limited literature reviewed in this field has primarily used a single HFUS method in mouse models of steatosis induced by highly unbalanced diets that bear little resemblance to the human Western diet and/or the animals were analyzed only at a late stage of the disease, raising scientific and/or animal welfare concerns. We have proposed a refined methodology demonstrating how a comprehensive and integrated approach to in vivo HFUS image analysis has the potential to significantly contribute to the early diagnosis and monitoring of hepatic steatosis in wild-type and genetically engineered mouse models that closely resemble the first, more easily reversible alterations occurring in human MASLD. These preliminary data suggest opportunities for best practices, as well as potential problems and future perspectives, and could be useful for further research aimed at validating disease progression models or studying therapeutic interventions. Importantly, ethical considerations were pursued throughout the research process, maximizing information on the various changes in liver characteristics using serial in vivo imaging, which allows for refinement of methods for detecting and monitoring steatosis and reduces the number of animals sacrificed in accordance with animal welfare guidelines.

This study has some positive attributes, including the following:

− Focused research questions;
− Realistic mouse model of early-stage DI-MASLD
− Translatable methodology;
− Noninvasive monitoring of pathological changes in the same animal, reducing biological variability;
− Multiparametric mouse phenotyping;
− Examination of multiple liver lobes to further increase accuracy and provide a more complete assessment of the extent of hepatic steatosis;
− Availability of a normal liver database from a control group (SD-fed WT mice);
− Timely histology after HFUS assessment at experimental endpoint;
− Blinded operators to each other’s analysis results;
− Use of DICOM format and proprietary tools or open-source software for image analysis.

## 5. Limitations and Challenges

Despite the strengths and promising observations, this study has several limitations. This is a preliminary retrospective study of an investigational technology, including a small number of animals. Inevitably, with a small, unbalanced sample size, statistical power is limited. Indeed, preclinical research must balance two conflicting needs in study design: (i) the use of the smallest justifiable sample size adhering to cost/benefit assessments, and the ethical dictates of regulatory rules; (ii) recruiting the most appropriate number of animals to apply inferential tests with adequate statistical power.

At the same time, proper statistical analysis of experimental data can help further reduce the number of animals by optimizing the information derived, thus increasing the overall scientific quality [73,74,77].

Although retrospective preclinical studies are usually conducted with very small sample sizes, with the aim of describing the main aspects and exploring the feasibility and challenges, some specific concerns of animal research need to be considered [77]. We calculated the sample size a priori, comparing means by extrapolating the expected data from the relevant scientific literature, based on the comparison of the effects of a high-fat diet in C57BL/6J mice and most commonly used diet-induced obesity GEMMs, like ob/ob or db/db mice [52]. However, diets administered in these studies are often more unbalanced than WD in terms of fat and carbohydrate content and/or administered for a longer time. Overall, these aspects of the experimental design could likely lead to a more evident effect size. Nonetheless, to our knowledge, no data are currently available on the effect of WD in MAPK15-ERK8 KO mice.

Because of the adjustments required for the analysis of multiple outcomes to avoid false positive errors, the lack of statistical significance among some HFUS variables assessed with respect to histological grade could be related to the low number of subjects within each sub-group [73,77].

Notably, in our study, GLCM texture feature analysis did not significantly improve diagnostic performance. Several limitations and difficulties, in addition to the small sample size, may have influenced this result, including the retrospective study design, the lack of standardized protocols in mouse models of steatosis, motion artifacts, or inter-operator variability in ROI selection. Although retrospective examination of imaging data has often been used in the initial exploration of texture analysis research, image acquisition parameters may not be optimized for quantitative feature analysis and may negatively impact the results.

However, exploratory investigations in animal models of MASLD may benefit from using descriptive statistics to analyze clinical, biochemical, imaging, and histological variables, providing a clear summary of key data. Measures such as mean, standard deviation, and percentages provide insights into disease patterns and progression, supporting the development of best practices, identification of potential limitations and possible solutions, extraction of as much useful information as possible from a dataset, and minimization of animal waste. These concerns highlight the need for improved methodologies to more effectively identify and follow the progression of this specific condition in murine models. Our findings may constitute a preliminary step towards further applied research on noninvasive tools capable of early detection and classification of fatty liver disease in mouse models using a comprehensive, translatable, and standardized HFUS approach.

Future prospective studies incorporating larger datasets, implementing respiratory gating or image post-processing to reduce motion effects, standardizing ROI selection through consensus expert annotations or automated segmentation tools, and implementing more advanced radiomics or deep learning techniques could potentially address these issues. These latest techniques have the potential to capture more subtle patterns that are not detected with conventional texture features such as GLCM and can overcome the challenges of inter-operator variability by automatically acquiring a larger number of features directly from the image data, without the need for manual feature selection or ROI definition. Importantly, they can further contribute to the principles of refinement and reduction in preclinical research, potentially extracting more information from the same experimental dataset.

Nevertheless, integrating complementary and evolving data, such as longitudinal changes in biomarker levels and imaging findings, is essential to improve the predictive capabilities of these models and algorithms. Furthermore, challenges remain in standardizing preclinical imaging protocols and ensuring accessibility to advanced imaging technologies in preclinical research.

Despite the potential impact of the animal stress and experimental design on blood biochemistry measurements of mice, in particular for glucose, there is poor standardization regarding experimental methodologies including fasting [78]. In rodent models, fasting blood glucose has been widely used to monitor the progression of diabetes, while postprandial blood glucose has been useful to highlight early changes in glucose metabolism [56]. Finally, this study involved a limited number of research institutions and HFUS acquisition was performed by a single operator using a single technological device, as was histology evaluation. Overall, these factors may affect the generalizability of the findings. Furthermore, although the proposed USICA approaches are computer-aided, they still require specialized imaging expertise, so reproducibility may depend on the operator’s skills. Further future studies are needed to enhance the feasibility of HFUS imaging methods to assess progression of hepatic steatosis in murine models, including larger datasets, a wide range of hepatic steatosis degrees, and scanners from different manufacturers. The methodology proposed has been easily implemented in research practice using HFUS scanner tools and open-source software for image post-processing. However, additional approaches, such as contrast-enhanced molecular US imaging, and further investigation of the complex interplay between imaging features and histopathology in larger cohorts, could contribute to a more comprehensive understanding of MASLD-associated liver pathologies in murine models.

## 6. Conclusions

In conclusion, our study provides an initial proof-of-concept to demonstrate the feasibility of an integrated HFUS method to assess the progression of hepatic steatosis in WT mice and GEMMs. We proposed combining CUS, QUS, and a CAD approach for the analysis of liver texture on conventional B-mode images acquired in the early stages of DI and clinical disease.

In the hands of experienced operators, the examined qualitative and quantitative US parameters had good performance for screening hepatic steatosis. Furthermore, the application of rigorous methodologies supports the postulate that the extracted imaging features predominantly reflect real differences in tissue samples related to different degrees of steatosis, avoiding significant procedural biases.

Based on our findings, HFUS may contribute to an improvement of the translational value of the research results in a more ethical way, by applying the principles of refinement and reduction. This technique can be used as a noninvasive tool to monitor the evolution of steatosis over time in mice, with promising implications in terms of therapeutic efficacy.

Implementation of simple HFUS image analysis approaches with QUS features could reduce the user-dependent limitation of this technique in the detection of early-stage hepatic steatosis in preclinical studies.

Although some parameters showed greater significance, our results highlight the value of a multiparametric approach, in which different imaging features contribute to disease identification and classification.

Future studies with larger samples and different mouse models could further standardize qualitative and quantitative US parameters and/or implement a more comprehensive assessment of hepatic steatosis with greater reliability and accuracy. Moreover, the diagnostic efficacy of certain imaging features, as a function of the disease under investigation, should be further analyzed. Radiomics continues to offer hope for early detection and improved stratification in MASLD models.

## Figures and Tables

**Table 1:** Descriptive statistics of body mass, food intake, feed efficiency, and serum biochemistry in WT and KO mice fed SD or WD.

Variables	Groups			
	WT SD (# 7)	WT WD (# 16)	KO SD (# 7)	KO WD (# 15)
Biometric and nutritional parameters				
Cumulative * BW gain ^†^, g/week	120.5 ± 15.5	151.4 ± 35.7	100.1 ± 18.7	147.5 ± 68.8
Cumulative * Energy intake, Kcal/day/mice	239.4 ± 18.1	262.8 ± 28.3	305.4 ± 72.9	358.8 ± 185.7
Cumulative * FER ^§^, %	122.7 ± 19.0	147.4 ± 38.1	90.5 ± 36.8	144.95 ± 80.3
Serum biochemistry °				
BUN (mg/dL)	55.16 ± 14.85	20.4 ± 5.0	69.7 ± 57.8	42.3 ± 10.5
Creatinine (mg/dL)	0.23 ± 0.07	0.19 ± 0.1 ^●^	0.37 ± 0.12	0.22 ± 0.1

Legend: # n: number of animals for each group. * means ± standard deviation after 17 weeks of diet per mouse in each group. ^†^ body weight (BW) change (g) from the initial body weight measurements. ^§^ food efficiency ratio (FER) was monitored weekly as the (total weight gain / total food intake) × 100. ° measurements at 24 weeks of age. BUN: Blood Urea Nitrogen. ^●^ mean value from # 8 WT WD mice.

**Table 2:** Descriptive statistics of non-fasting cholesterol, triglycerides, and glucose from 8 (baseline, pre-diet) to 16 weeks of age in WT and KO mice fed SD or WD.

	Groups							
	WT SD (# 7)		WT WD (# 16)		KO SD (# 7)		KO WD (# 15)	
Age (Weeks)	8	16	8	16	8	16	8	16
Variables								
Cholesterol (mg/dL)	148.7 ± 25.8	150.0 ± 9.5	146.8 ± 18.3	197.2 ± 34.9	151.3 ± 18.8	142.6 ± 18.8	146.7 ± 11.8	194.1 ± 28.5
Triglycerides (mg/dL)	132.7 ± 21.2	214.7 ± 43.0	190.2 ± 62.4	116.4 ± 31.6	297.0 ± 63.1	232.0 ± 72.7	182.2 ± 59.3	109.7 ± 35.3
Glucose (mg/dL)	159.7 ± 11.9	166.4 ± 21.9	176.8 ± 33.0	170.7 ± 22.0	133.4 ± 17.4	180.4 ± 22.7	167.2 ± 27.5	196.2 ± 24.9

# n: number of animals for each group.

**Table 3:** Histological severity of hepatic steatosis related with the CUS score in 24-week-old WT and KO mice fed SD or WD.

		Score of Steatosis in Histological Sample							
	CUS	0 (#7)	1 (#0)	2 (#0)	3 (#0)	4 (#0)	5 (#0)	6 (#0)	7 (#0)
WT SD (# 7)	0	6 (86%)	0	0	0	0	0	0	0
	1	1 (14%)	0	0	0	0	0	0	0
	2	0	0	0	0	0	0	0	0
	3	0	0	0	0	0	0	0	0
	4	0	0	0	0	0	0	0	0
	5	0	0	0	0	0	0	0	0
	6	0	0	0	0	0	0	0	0
		0 (#4)	1 (#0)	2 (#2)	3 (#1)	4 (#0)	5 (#0)	6 (#0)	7 (#0)
KO SD (# 7)	0	3 (43%)	0	0	0	0	0	0	0
	1	0	0	1 (14.25%)	0	0	0	0	0
	2	1(14.25%)	0	1 (14.25%)	1 (14.25%)	0	0	0	0
	3	0	0	0	0	0	0	0	0
	4	0	0	0	0	0	0	0	0
	5	0	0	0	0	0	0	0	0
	6	0	0	0	0	0	0	0	0
		0 (#4)	1 (#4)	2 (#4)	3 (#3)	4 (#0)	5 (#0)	6 (#0)	7 (#1)
WT WD (# 16)	0	0	0	0	0	0	0	0	0
	1	4 (25%)	3 (18.75%)	1 (6.25%)	0	0	0	0	0
	2	0	1 (6.25%)	3 (18.75%)	2 (12.5%)	0	0	0	0
	3	0	0	0	0	0	0	0	0
	4	0	0	0	1 (6.25%)	0	0	0	0
	5	0	0	0	0	0	0	0	1 (6.25%)
	6	0	0	0	0	0	0	0	0
		0 (#0)	1 (#1)	2 (#2)	3 (#2)	4 (#2)	5 (#3)	6 (#4)	7 (#1)
KO WD (# 15)	0	0	0	0	0	0	0	0	0
	1	0	1 (6.7%)	0	0	0	0	0	0
	2	0	0	0	0	0	0	0	0
	3	0	0	1 (6.7%)	1 (6.7%)	1 (6.7%)	1 (6.7%)	1 (6.7%)	1 (6.7%)
	4	0	0	1 (6.7%)	1 (6.7%)	0	1 (6.7%)	3 (19.6%)	0
	5	0	0	0	0	1 (6.7%)	1 (6.7%)	0	0
	6	0	0	0	0	0	0	0	0

Legend: (# n): number of animals for each histological score per group. n (%): number of animal (and relative percentage) for each ultrasonographic score per group.

**Table 4:** Histological severity of hepatic steatosis related with the QUS estimates in 24-week-old WT and KO mice fed SD or WD.

		Score of Steatosis in Histological Sample							
	QUS	0 (# 7)	1 (# 0)	2 (# 0)	3 (# 0)	4 (# 0)	5 (# 0)	6 (# 0)	7 (# 0)
WT SD (# 7)	HRI	0.69 ± 0.1 (0.5–0.8)	0	0	0	0	0	0	0
	no steatosis	0.69 ± 0.1 (0.5–0.8)							
	steatosis	NP							
		0 (#4)	1 (#0)	2 (#2)	3 (#1)	4 (#0)	5 (#0)	6 (#0)	7 (#0)
KO SD (# 7)	HRI	0.68 ± 0.07 (0.6–0.8)	0	0.81 ± 0.14 (0.7–0.9)	0.77	0	0	0	0
	no steatosis	0.68 ± 0.07 (0.6–0.8)							
	steatosis	>0.8							
		0 (#4)	1 (#4)	2 (#4)	3 (#3)	4 (#0)	5 (#0)	6 (#0)	7 (#1)
WT WD (# 16)	HRI	0.74 ± 0.05 (0.7–0.8)	0.73 ± 0.07 (0.7–0.8)	0.91 ± 0.1 (0.6–1.1)	0.99 ± 0.05 (0.9–1.1)	0	0	0	1.25
	no steatosis	0.74 ± 0.05 (0.7–0.8)							
	steatosis	>0.8							
		0 (#0)	1 (#1)	2 (#2)	3 (#2)	4 (#2)	5 (#3)	6 (#4)	7 (#1)
KO WD (# 15)	HRI	0	0.73	0.8 ± 0.12 (0.7–0.9)	0.7 ± 0.08 (1.0–1.1)	1.07 ± 0.1 (1.0–1.1)	0.97 ± 0.3 (0.6–1.2)	1.25 ± 0.2 (1.1–1.6)	0.85
	no steatosis	NP							
	steatosis	>0.7							

Legend: (# n): number of animals for each histological score per group. n ± n^2^ (n^3^–n^4^): mean ± standard deviation (and relative range) of ultrasonographic feature for each histological score per group. NP: not present.

## Data Availability

The original contributions presented in this study are included in the article and supplementary material. Further inquiries can be directed to the corresponding author.

## Supplementary Materials

The following supporting information can be downloaded at: https://www.mdpi.com/article/10.3390/jimaging11100369/s1.
